# ^68^Ga-NOTA-Evans Blue PET/CT findings in lymphangioleiomyomatosis compared with ^99m^TC-ASC lymphoscintigraphy: a prospective study

**DOI:** 10.1186/s13023-021-01895-1

**Published:** 2021-06-16

**Authors:** Guozhu Hou, Yuanyuan Jiang, Wenshuai Xu, Zhaohui Zhu, Li Huo, Xiaoyuan Chen, Fang Li, Kai-Feng Xu, Wuying Cheng

**Affiliations:** 1 Department of Nuclear Medicine, State Key Laboratory of Complex Severe and Rare Diseases, Peking Union Medical College Hospital, Chinese Academy of Medical Sciences and Peking Union Medical College, Beijing, 100730 China; 2 Beijing Key Laboratory of Molecular Targeted Diagnosis and Therapy in Nuclear Medicine, Beijing, 100730 China; 3 Department of Respiratory Medicine, State Key Laboratory of Complex Severe and Rare Diseases, Peking Union Medical College Hospital, Chinese Academy of Medical Sciences and Peking Union Medical College, Beijing, 100730 China; 4 Departments of Diagnostic Radiology, Chemical and Biomolecular Engineering, and Biomedical Engineering, National University of Singapore, Singapore, 117545 Singapore; 5 Department of Nuclear Medicine, Peking Union Medical College Hospital, Chinese Academy of Medical Sciences and Peking Union Medical College, Beijing, 100730 China; 6 Department of Respiratory Medicine, Peking Union Medical College Hospital, Chinese Academy of Medical Sciences and Peking Union Medical College, Beijing, 100730 China

**Keywords:** Lymphangioleiomyomatosis, ^68^Ga-NOTA-Evans Blue (^68^Ga-NEB), PET/CT, Lymphoscintigraphy

## Abstract

**Background:**

Lymphangioleiomyomatosis (LAM) is a rare multisystem disease characterized by cystic lung disease and extrapulmonary manifestations, including lymphatic system disorder. The objective of this study was to investigate the findings of ^68^Ga-NOTA-Evans Blue (NEB) PET/CT in LAM and compare it with that of ^99m^Tc-ASC lymphoscintigraphy.

**Methods:**

Ten patients diagnosed with LAM according to the American Thoracic Society/Japanese Respiratory Society guidelines for LAM were recruited in this study. PET/CT acquisition was performed at 20 to 40 min after subcutaneous injection of ^68^Ga-NEB into the first interdigital spaces of both feet (0.3 ml, 37 MBq/foot). All subjects also underwent ^99m^Tc-antimony sulfide colloid (ASC) lymphoscintigraphy within a week for comparison.

**Results:**

^68^Ga-NEB PET/CT displayed various lymphatic system abnormalities in 10 (100%) of 10 patients. These included pulmonary lymphatic abnormalities in 10 (100%) of 10 patients, enlarged lymph nodes in 5 (50%), lymphangioleiomyomas in 2 (20%), dilation of the lumbar trunk and/or iliac lymph vessels in 5 (50%), thoracic duct dilation in 2 (20%), chylous effusion in 1 (10%). For pulmonary lymphatic abnormalities, the positive rates of ^68^Ga-NEB PET/CT and ^99m^Tc-ASC lymphoscintigraphy were 100% (10/10) and 10% (1/10), respectively (*P* < 0.001). As for the 7 patients with extrapulmonary lymphatic manifestations, ^68^Ga-NEB PET/CT also presented more information than ^99m^Tc-ASC lymphoscintigraphy.

**Conclusion:**

^68^Ga-NEB PET/CT visualized pulmonary lymphatic abnormality and displayed extrapulmonary lymphatic system disorders of LAM, and might play a role in the diagnosis and evaluation of the disease. ^68^Ga-NEB PET/CT is advantageous over conventional ^99m^Tc-ASC lymphoscintigraphy in LAM by providing more detailed information of lymphatic dysfunction.

## Introduction

Lymphangioleiomyomatosis (LAM), a rare multi-system disease primarily found in women, is characterized by diffuse cystic changes in the lung [1, 2]. LAM mainly affects the lung, but can also involve the thoracic and abdominal axial lymphatics, including the lymph nodes in the pelvic cavity, retroperitoneum, mediastinum, and thoracic duct [3]. LAM lesions are generated by the proliferation of immature smooth muscle-like cells (LAM cells) [4]. Patients with LAM may present with dyspnea, chylous pleural effusion, pneumothorax, hemoptysis, and symptoms associated with extrapulmonary involvement [5]. Extrapulmonary manifestations, occurring in more than 70% of patients, include angiomyolipomas (AMLs), lymphangioleiomyomas, lymphadenopathy, and lymphatic dilation [6].

A previous retrospective study used CT lymphangiography to evaluate the lymphatic system disorder in 27 patients with LAM and observed various axial lymphatic system manifestations in the thorax and abdomen [7]. However, nuclear medicine imaging findings of the lymphatic system disorder in LAM patients have not been described comprehensively before. ^99m^Tc-antimony sulfide colloid (ASC) lymphoscintigraphy is a widely used method for lymphatic mapping. Until now, there has been no report of ^99m^Tc-ASC lymphoscintigraphy findings of LAM. ^68^Ga-NOTA Evans Blue (^68^Ga-NEB) is an albumin-binding PET radiotracer for lymphatic imaging and has been used in several lymphatic disorders for diagnosis and evaluation [8–14]. We recently reported a LAM patient whose ^68^Ga-NEB PET/CT not only clearly displayed the lymphatic disorders in the abdomen but also unexpectedly revealed diffuse abnormal NEB activity in bilateral lungs, suggesting the existence of pulmonary lymphatic circulation abnormality [15]. Therefore, in this current prospective study, we aimed to further evaluate ^68^Ga-NEB PET/CT in LAM and to compare it with ^99m^Tc-ASC lymphoscintigraphy.

## Patients and methods

### Patients

This study was part of the study “Application of ^68^Ga-NEB PET Imaging in the Diagnosis and Evaluation of Lymphatic Disorders” registered at clinicaltrials.gov (NCT 02496013) and approved by the Institute Review Board of Peking Union Medical College Hospital (PUMCH) (IRB protocol #ZS-2131).

A total of 10 patients (10 women, aged 21–50 years [37.40 ± 10.10 years]]) diagnosed with LAM according to the American Thoracic Society/Japanese Respiratory Society guidelines [16] for LAM were consecutively recruited from October 2019 to May 2020. All patients were recommended by the Department of Pulmonary and Critical Care Medicine. The exclusion criteria were patients (1) with mental illness; (2) with severe liver or kidney dysfunction; (3) with hematopoietic dysfunction; (4) who were pregnant or breast-feeding. All patients underwent both ^68^Ga-NEB PET/CT and ^99m^Tc-ASC scintigraphy within 1 week. Statement of informed consent was obtained from all patients included in the study.

### ^68^Ga-NEB PET/CT study

The ^68^Ga-NEB was produced following our previously published procedure [9]. ^68^Ga-NEB was subcutaneously injected into the first interdigital spaces of both feet (0.3 mL, 37 MBq/foot). The patients were requested to walk after tracer injection. 20–40 min later, PET scan (7–10 bed positions, 2 min/bed) covering from the feet to the neck was acquired after a low-dose CT scan (120 keV; 100 mAs; 1.3 pitch; 2.5 mm slice thickness; 0.5 s rotation time; estimated radiation dose 9.0 mGy). The acquired images were reconstructed using the ordered subsets expectation–maximization (OSEM) algorithm (2 iterations, 10 subsets,Gaussian filter, 192 × 192 matrix).

### ^99m^Tc-ASC lymphoscintigraphy

All patients underwent ^99m^Tc-ASC lymphoscintigraphy for comparison within a week of ^68^Ga-NEB PET/CT. The lymphoscintigraphy acquisition was performed at 1 h after ^99m^Tc-ASC was subcutaneously injected into first and second interdigital spaces of both feet (0.5 mL, 37 MBq/foot). Images were acquired with a double-head gamma camera and a low-energy high-resolution parallel whole collimator in whole-body scanning mode with a 256 × 1024 matrix at a scan speed of 15 cm/min.

### Image interpretation and statistical analysis

Visual analysis was applied for image interpretation. The images were read by two experienced nuclear medicine physicians. The criteria of the visual analysis were as follows: (1) visualization of pulmonary lymphatic abnormality (yes/no) and the distribution of tracer uptake; (2) dilated lymphatic vessels (yes/no) and sites; (3) the presence and locations of lymphadenopathy; (4) lymphangioleiomyomas (yes/no) and distribution of tracer; (5) chylous effusion (yes/no). Data were expressed as mean ± SD. Statistical analyses were done with the SPSS Statistics software (version 24.0, IBM SPSS Inc.). The Chi-squared test was used to compare the positive rates of ^68^Ga-NEB PET/CT and ^99m^Tc-ASC lymphoscintigraphy. The *P*-value < 0.05 was considered statistically significant.

## Results

### ^68^Ga-NEB PET/CT findings

**Fig. 1:**
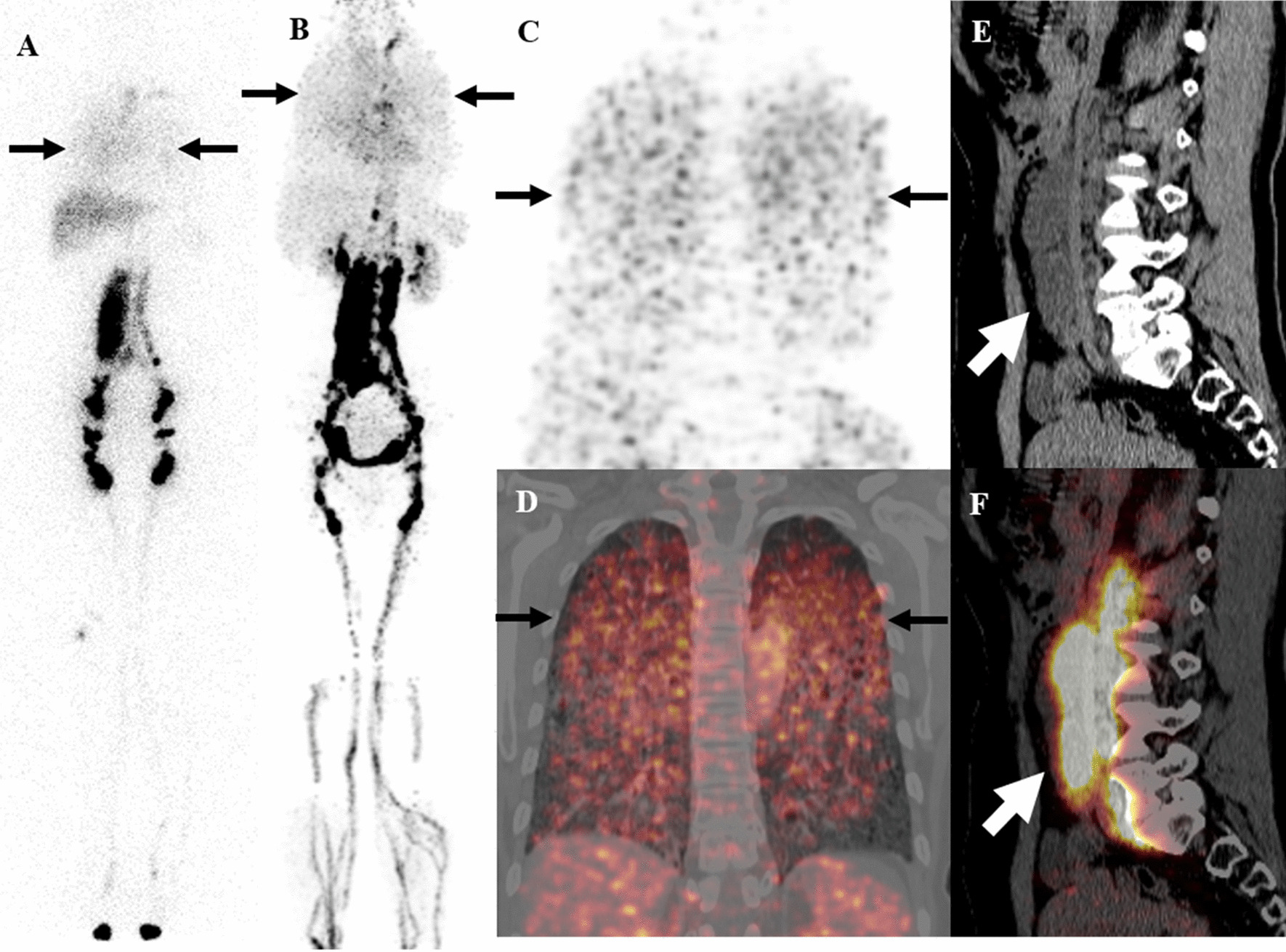
A 50-year-old woman (patient #**2**) with LAM. Both ^99m^Tc-ASC lymphoscintigraphy (**A**, black arrows) and ^68^Ga-NEB PET/CT MIP image (**B**, black arrows) showed diffuse, increased activity in the chest and intense vertical activity in the abdomen. Coronal images (**C**, PET; **D**, fusion; black arrows) of ^68^Ga-NEB PET/CT of the chest demonstrated that the abnormal chest activity was diffusely distributed in the lung, suggesting diffuse pulmonary lymphatic changes. On sagittal images (**E**, CT; **F**, fusion; white arrows) of ^68^Ga-NEB PET/CT of the abdomen, the vertical activity was located in the dilated lumbar trunk

^68^Ga-NEB PET/CT displayed various abnormal lymphatic system manifestations in all 10 patients (100%) with LAM. Abnormally increased NEB activity in the lung was observed in 10 of 10 (100%) patients. The NEB activity was diffusely distributed in bilateral lungs (Fig. 1, patient #**2**).

**Fig. 2:**
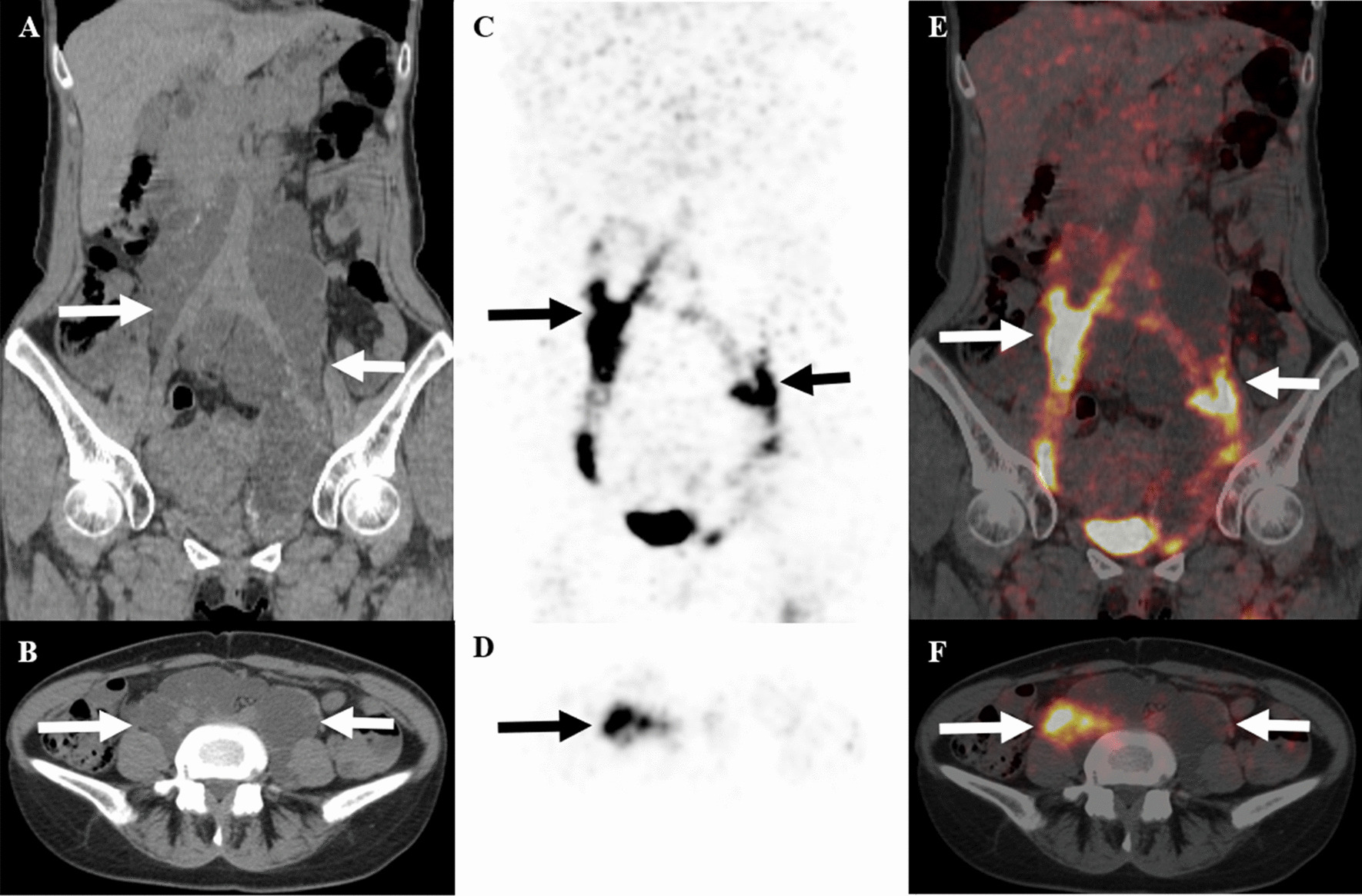
A 48-year-old woman (patient #**8**) with LAM. Co-registered CT scans (**A**, coronal view, **B**: axial view; white arrows) of ^68^Ga-NEB PET/CT demonstrated a large hypodense multiloculated cystic mass extending from the retroperitoneum into the pelvis, consistent with lymphangioleiomyoma. On ^68^Ga-NEB PET (**C,** coronal view; **D**, axial view; black arrows) and fusion (**E**, coronal view; **F**, axial view; white arrows) images, the tracer was only seen in part of the cystic components

Seven (70%) of 10 patients were presented with extrapulmonary lymphatic manifestations on ^68^Ga-NEB PET/CT. Multiple enlarged lymph nodes were found in 5 (50%) of 10 patients with intense NEB activity on PET/CT images. The sizes ranged from 1.0 to 1.9 cm. These nodes were retroperitoneal and pelvic in 2 patients, and were retroperitoneal in 3 patients. ^68^Ga-NEB PET/CT showed retroperitoneal and pelvic lymphangioleiomyomas in 2 (20%) patients, which were presented as hypoattenuating partially cystic and partially solid mass, measuring 23.9 cm and 4.2 cm at maximum diameter, respectively. NEB accumulation was observed in part of the cystic components (Fig. 2, patient #**8**). Dilation of the thoracic segment of the thoracic duct was noted in 2 patients (20%), measuring 1.4 and 1.6 cm in diameter, respectively. 5 patients (50%) had retroperitoneal lumbar trunk and/or iliac lymphatic vessel dilation, with sizes ranging from 0.5 to 3.5 cm. Abdominopelvic chylous effusion was observed in one patient (10%), demonstrating mild NEB activity.

### Comparison between ^99m^Tc-ASC lymphoscintigraphy and ^68^Ga-NEB PET/CT findings

**Fig. 3:**
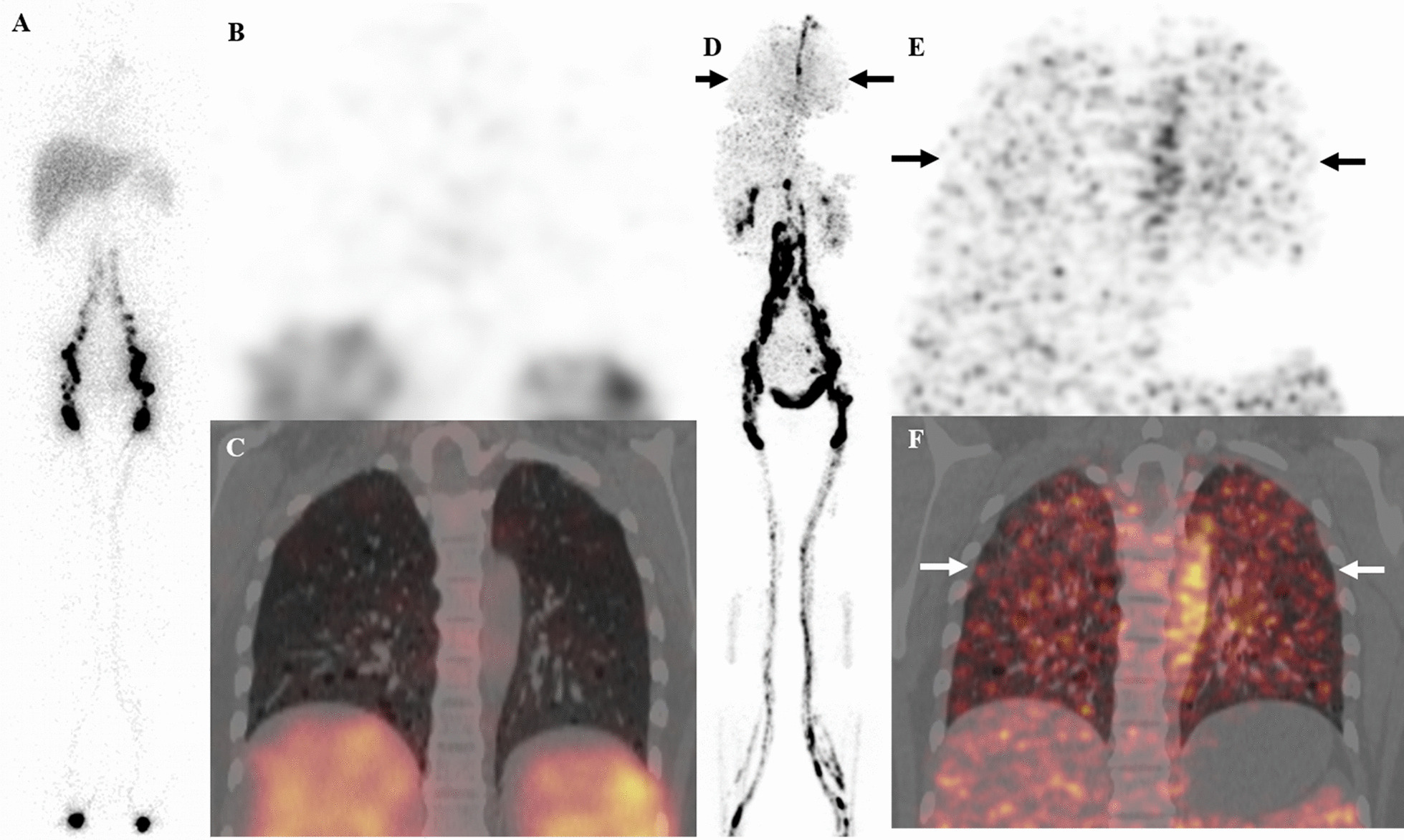
A 34-year-old woman (patient #**7**) with LAM. The ^99m^Tc-ASC lymphoscintigraphy (**A**) and SPECT/CT (**B**, SPECT; **C**, fusion) at 1 h after tracer injection appeared negative. However, on ^68^Ga-NEB MIP image (**D**, arrows), there was abnormal, increased activity in the chest. And coronal images (**E**, PET; **F**, fusion; arrows) of the chest revealed that the chest activity was in the lung. No extrapulmonary lymphatic manifestations were found on ^68^Ga-NEB PET/CT

**Fig. 4:**
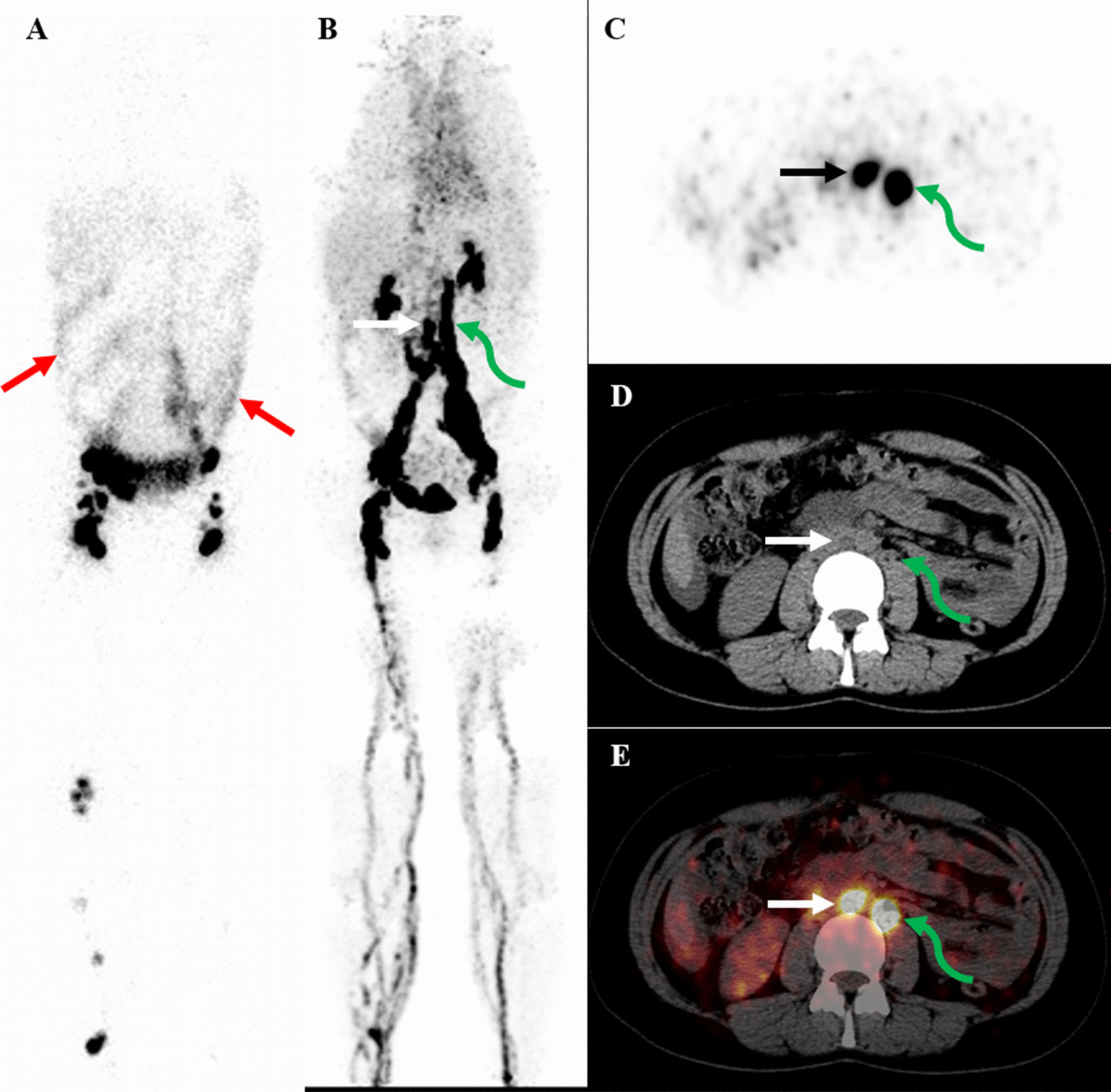
A 30-year-old woman (patient #**5**) with LAM. The patient had ascites and laboratory examination indicated chyloperitoneum. ^99m^Tc-ASC lymphoscintigraphy (**A**, red arrows) showed mild diffuse activity in the abdomen with barely no chest activity. ^68^Ga-NEB PET/CT images revealed abnormal activity in the chest (**B,** MIP) and retroperitoneal lymphatic vessel dilation (white arrows) and lymphadenopathy (green arrows) (**C**, PET; **D**, CT; **E**, fusion). Interestingly, the abnormal activity in the ascites was not as obvious on ^68^Ga-NEB PET/CT images as on ^99m^Tc-ASC lymphoscintigraphy

Four (40%) of 10 patients showed positive findings on ^99m^Tc-ASC lymphoscintigraphy. For pulmonary lymphatic abnormality, ^68^Ga-NEB PET/CT was positive in all 10 patients (100%), while ^99m^Tc-ASC lymphoscintigraphy was only positive in 1 patient (10%, *P* < 0.001; Fig. 3, patient #**7**). For extrapulmonary lymphatic system abnormalities, the results of ^99m^Tc-ASC lymphoscintigraphy and ^68^Ga-NEB PET/CT were consistent in 1 patient (Patient #**2**), while ^68^Ga-NEB PET/CT revealed more lesions than ^99m^Tc-ASC lymphoscintigraphy in 6 patients. Compared to ^99m^Tc-ASC lymphoscintigraphy, ^68^Ga-NEB PET/CT showed added value in 9/10 patients for pulmonary lymphatic abnormalities, 5/10 patients for enlarged lymph nodes, 3/10 patients for dilation of the lumbar trunk and/or iliac lymph vessels, 1/10 patients for thoracic duct dilation and 1/10 patients for lymphangioleiomyomas. In patient #**1** and #**9**, ^99m^Tc-ASC lymphoscintigraphy was unable to show the retroperitoneal lymph nodes, which were detected by ^68^Ga-NEB PET/CT. In patient #**4**, ^68^Ga-NEB PET/CT revealed multiple lymphatic abnormalities including retroperitoneal lymph nodes and lymphatic vessel dilation, thoracic duct dilation, and pelvic lymphangioleiomyoma while ^99m^Tc-ASC lymphoscintigraphy appeared normal. In patient #**5** and #**10**, ^68^Ga-NEB PET/CT provided more information than ^99m^Tc-ASC lymphoscintigraphy by visualizing retroperitoneal lumbar trunk dilation and/or enlarged lymph nodes (Fig. 4, patient #**5**). Chyloperitoneum with mild tracer activity was found on both ^68^Ga-NEB PET/CT and ^99m^Tc-ASC lymphoscintigraphy. In patient #**8**, ^68^Ga-NEB PET/CT showed multiple lymph nodes in the retroperitoneum and pelvis which ^99m^Tc-ASC lymphoscintigraphy was unable to visualize (Table 1).

**Table 1:** ^68^Ga-NEB PET/CT and ^99m^TC-ASC lymphoscintigraphy findings in patients with LAM

Patient	Age/sex	Pulmonary lymphatic abnormalities		Enlarged lymph nodes		Dilation of lumbar trunk and/or iliac lymph vessels		Thoracic duct dilation		Lymphangioleiomyomas		Chylous effusion	
		NEB	ASC	NEB	ASC	NEB	ASC	NEB	ASC	NEB	ASC	NEB	ASC
**1**	34/F	***Y***	*N*	***Y***	*N*	N	N	N	N	N	N	N	N
**2**	50/F	**Y**	**Y**	N	N	**Y**	**Y**	N	N	N	N	N	N
**3**	48/F	***Y***	*N*	N	N	N	N	N	N	N	N	N	N
**4**	33/F	***Y***	*N*	***Y***	*N*	***Y***	*N*	***Y***	*N*	***Y***	*N*	N	N
**5**	30/F	***Y***	*N*	***Y***	*N*	***Y***	*N*	N	N	N	N	**Y**	**Y**
**6**	29/F	***Y***	*N*	N	N	N	N	N	N	N	N	N	N
**7**	34/F	***Y***	*N*	N	N	N	N	N	N	N	N	N	N
**8**	48/F	***Y***	*N*	***Y***	*N*	N	N	N	N	**Y**	**Y**	N	N
**9**	47/F	***Y***	*N*	***Y***	*N*	**Y**	**Y**	**Y**	**Y**	N	N	N	N
**10**	21/F	***Y***	*N*	N	N	***Y***	*N*	N	N	N	N	N	N

The bold (Y) indicate that the imaging results are positive. The “Y-N” pairs where NEB would be positive and ASC would be negative appear in italics. The “Y” of the “Y–N” pairs where NEB would be positive and ASC would be negative appear in bold italics

### Extralymphatic system abnormality

Multiple round thin-walled air-filled cysts in the lung characteristic of LAM have been observed in all 10 patients on ^68^Ga-NEB PET/CT. 1 (10%) of 10 patients presented with 2 renal AMLs, with a size of 0.6 and 1.2 cm, respectively.

## Discussion

LAM is characterized by the proliferation of LAM cells in the affected organs. LAM cells can produce VEGF-D, a lymphangiogenic growth factor [17]. It is speculated that VEGF-D promotes the local aggregation of lymphatic endothelial cells, which then promotes the formation of lymphatics [18]. Extensive lymphatic vessels in both pulmonary and extrapulmonary LAM lesions have already been noted and described as cystic or slit-like spaces within the LAM foci in histopathologic studies [19–22]. Our finding of diffuse NEB activity in the lung on PET/CT images, suggesting the hyperplasia and dilation of pulmonary lymphatic vessels, supported the findings of these histopathologic studies. Obviously, ^68^Ga-NEB PET/CT is an ideal imaging method to visualize the existence of extensive lymphatic changes in the lung region.

The existence of pulmonary lymphatic abnormality in LAM is not fully acknowledged in the clinic, which is attributed to the fact that in clinical practice, the examination of lymphatic vessels in lung specimen is not routinely performed as the presence of lymphatic vessel within LAM foci is not required for the pathologic diagnosis of LAM [23]. According to the American Thoracic Society/Japanese Respiratory Society guidelines, a definite diagnosis of LAM can be made based on the presence of cystic changes on HRCT of the chest characteristic of LAM and any of the following confirmatory features: renal AML, chylous effusion, lymphangioleiomyoma, adenopathy, lymphatic vessels dilation, and either definite or probable tuberous sclerosis complex (TSC) [23]. If these extrapulmonary features of LAM are not evident, a lung biopsy would be required to confirm the diagnosis. Since we were able to visualize pulmonary lymphatic abnormality by ^68^Ga-NEB PET/CT, pulmonary lymphatic abnormality might be considered as one additional confirmatory feature of LAM to aid the diagnosis and evaluation of the disease. With ^68^Ga-NEB PET/CT, the confidence of diagnosis in patients, especially those found with only pulmonary cystic changes would be increased.

Extrapulmonary lymphatic system manifestations of LAM observed in our study included adenopathy, lymphatic vessel dilation, retroperitoneal and pelvic lymphangioleiomyoma, and chylous effusion. As mentioned above, these extrapulmonary lymphatic system disorders are confirmatory features of LAM, and the demonstration of these abnormalities with ^68^Ga-NEB PET/CT may aid the diagnosis and evaluation of the disease. Lymph adenopathy and lymphatic vessel dilation, generally presented with intense NEB activity, could be easily visualized on PET/CT images.

We also noticed that in lymphangioleiomyoma, manifested as a well-circumscribed multilocular mass with central fluid rich region on CT scan, NEB accumulation was only seen in part of the cystic components. It was reported that lymphangioleiomyoma is a result of smooth muscle cell proliferation in the lymph vessels, which then causes dilatation and obstruction in the lymph vessels and collection of chylous material [20, 24–26]. Therefore, part of the cystic components may communicate with the lymphatic system, which leads to tracer accumulation.

The positive rate of ^68^Ga-NEB PET/CT in detecting pulmonary lymphatic disorder is significantly higher than that of ^99m^Tc-ASC lymphoscintigraphy (*P* < 0.001). Considering that NEB binds to albumin during circulation and the size of NEB/albumin complex is much smaller than ^99m^Tc-ASCs, it is thus easier for NEB to reach the involved pulmonary lymph vessels, which are generally very small in diameter [13, 14, 19]. A previous study reported that CT lymphangiography showed intrapulmonary lymphatic vessel dilation in 11% (3/27) LAM patients [7]. It is also worth noting that the size of the observed dilated intrapulmonary lymphatic vessels ranged from 0.1 to 0.4 cm, raising the possibility that CT lymphangiography is able to detect relatively large lymphatic vessels but not small ones, which might explain the low positive rate (11%) of CT lymphangiography. Further studies of comparing ^68^Ga-NEB PET/CT and CT lymphangiography in evaluating LAM in more patients are need to confirm this finding.

In this study, ^68^Ga-NEB PET/CT is also more revealing than ^99m^Tc-ASC lymphoscintigraphy by presenting more extrapulmonary lymphatic disorders. Our results demonstrated that, compared to ^99m^Tc-ASC lymphoscintigraphy, ^68^Ga-NEB PET/CT showed added value in 9/10 patients for the detection of pulmonary lymphatic abnormalities, 5/10 patients for enlarged lymph nodes, 3/10 patients for dilation of the lumbar trunk and/or iliac lymph vessels, 1/10 patients for thoracic duct dilation and 1/10 patients for lymphangioleiomyomas. Similarly, a previous report also showed that ^68^Ga-NEB PET/CT presented more clinically important information than did ^99m^Tc-ASC lymphoscintigraphy in patients with lymphedema or chylous leakages [14]. This might be due to the fact that ^99m^Tc-ASC lymphoscintigraphy has an intrinsic disadvantage compared with ^68^Ga-NEB PET/CT. PET has greater intrinsic sensitivity compared to SPECT, and ^68^Ga-NEB PET has better spatial resolution than ^99m^Tc-ASC lymphoscintigraphy. In addition, ^68^Ga-NEB PET/CT images are dynamic 3-dimensional whereas traditional ^99m^Tc-ASC lymphoscintigraphy acquires only static 2-dimensional images. ^68^Ga-NEB PET/CT is also advantageous over ^99m^Tc-ASC lymphoscintigraphy in shorter waiting and acquisition time.

Lymphatic system abnormality has always been considered as complications of LAM and is reported to be found in about 20% of LAM patients [27, 28]. In our study, 70% (7/10) patients presented with extrapulmonary lymphatic manifestations on ^68^Ga-NEB PET/CT. However, the demonstration of the existence of pulmonary lymphatic changes with ^68^Ga-NEB PET/CT increases the proportion of cases with lymphatic involvement in our population to 100%. Based on this finding, we speculate that LAM patients who were diagnosed with only pulmonary cystic changes in the past might also have lymphatic involvement which conventional imaging methods failed to detect. Our finding of a high proportion of LAM cases with lymphatic involvement combined with the findings of histopathological studies suggest that lymphatic dysfunction may be a key mechanism in LAM pathogenesis [19–22]. Elucidation of the role of lymphatic dysfunction in LAM may have the potential to develop new therapies targeting lymphatic circulation to inhibit the progression of LAM [19]. Therefore, a great deal remains to be learned about lymphatic involvement in LAM, including its role in pathogenesis of the disease and its potential as a treatment target.

Several limitations of this study must be pointed out. First, the study is limited by the lack of histopathologic correlation of ^68^Ga-NEB PET/CT findings of pulmonary lymphatic changes. The second limitation is the small sample size of 10 patients with LAM. In future studies, we will collect more patients with LAM to further investigate the role of ^68^Ga-NEB PET/CT in assessing severity degree, treatment response, and predicting the prognosis of the disease. Thirdly, ^68^Ga-NEB PET/CT is able to visualize the pulmonary lymphatic abnormality and may have the potential in separating LAM from other cystic lung diseases such as emphysema, Langerhans cell histiocytosis, Sjögren syndrome with cystic changes in lung, and Birt-Hogg-Dubé syndrome. However, the current study did not examine the findings of ^68^Ga-NEB PET in other cystic lung diseases. In the future, studies investigating the ^68^Ga-NEB PET/CT findings of other cystic lung diseases were required to confirm this. In addition, we did not make correlations between VEGF-D level and the degree of lymphatic abnormality with ^68^Ga-NEB PET/CT in this study because VEGF-D data is not available in every patient. Besides, the sample size of 10 patients is too small, making it difficult to grade the severity of lymphatic abnormality with ^68^Ga-NEB PET/CT. In the future, we will collect more patients to grade the severity of lymphatic abnormality with ^68^Ga-NEB PET/CT, and to correlate VEGF-D level with ^68^Ga-NEB PET/CT findings.

## Conclusions

^68^Ga-NEB PET/CT demonstrated pulmonary lymphatic abnormality and displayed various extrapulmonary lymphatic system disorders of LAM, making it a promising method in the diagnosis and evaluation of the disease. The presence of pulmonary lymphatic abnormality, if added in the confirmatory features of LAM, may aid in the diagnosis of the disease. Compared with ^99m^Tc-ASC lymphoscintigraphy, ^68^Ga-NEB PET/CT is a more accurate method in evaluating LAM by providing more information.

## Data Availability

The datasets used and analyzed during the current study area available from the corresponding author.

## Abbreviations

LAM: Lymphangioleiomyomatosis
NEB: NOTA-Evans Blue
ASC: Antimony sulfide colloid
TSC: Tuberous sclerosis complex
AMLs: Angiomyolipomas
PET/CT: Positron emission tomography/computed tomography
SPECT/CT: Single photon emission computed tomography/computed tomography
